# A case of chronic disseminated candidiasis in metamizole-induced neutropaenia

**DOI:** 10.1007/s15010-022-01963-z

**Published:** 2022-12-14

**Authors:** Eugénie Colin-Benoit, Malela Kalubi, Stefan Zimmerli

**Affiliations:** Department of Infectious Diseases, Inselspital, Bern University Hospital, University of Bern, 3010 Bern, Switzerland

**Keywords:** Chronic disseminated candidiasis, Metamizole-induced neutropaenia, Immune reconstitution inflammatory syndrome, Corticosteroid therapy

## Abstract

Chronic disseminated candidiasis (CDC) is a severe complication of a disseminated yeast infection mainly seen after prolonged chemotherapy-induced neutropaenia in the context of haematological malignancy. We present a case of CDC in a patient with metamizole-induced neutropaenia. To the best of our knowledge, this is the first case described in this context. Furthermore, we highlight the role of steroids in the management of this disease.

## Case report

An 18-year-old woman was admitted with fever, odynodysphagia worsening over a few days and neutropaenia. The patient reported no B symptoms or other symptoms. Her past medical history was unremarkable; she smoked approximately five cigarettes/day but did not consume alcohol or illegal drugs. She used levonorgestrel and ethinylestradiol for contraception and metamizole, at least 500 mg/d for the last 3 months, to treat holocephalic headaches. Because of the COVID-19 pandemic and lockdown, the prescription was renewed several times by the family doctor by telephone but without follow-up consultation. On admission, the patient was hypotensive (85/55 mmHg), tachycardic (140/min) and febrile (39.1 °C), with bilateral tonsillitis, bilateral angular lymphadenitis and trismus. In addition to bilateral tonsillitis, a CT scan showed diffuse pharyngeal inflammation as well as phlegmonous changes in the submandibular soft tissues, with thickening of the left platysma. Neck abscesses and thrombosis of the jugular vein were ruled out. A full blood count revealed neutropaenia (leucocytes 0.25 G/L, exclusively lymphocytes), microcytic and hypochromic anaemia (haemoglobin 107 g/L, mean corpuscular volume 75 fL, mean corpuscular haemoglobin 25 pg) and a normal thrombocyte count (458 G/L). No blasts were identified. C-reactive protein (CRP) was elevated to 336 mg/L. Renal and liver function tests were normal, except for slightly elevated gamma-glutamyl transferase (77 U/L). Albumin was low (23 g/L), and the international normalized ratio (INR) was 1.37. A PCR test for SARS-CoV-2 using a nasopharyngeal swab was negative. She also tested negative for human immunodeficiency virus (HIV).

A diagnosis of sepsis originating from tonsillitis in the context of neutropaenia was made, and antibiotic therapy with piperacillin/tazobactam 3 × 4.5 gr/d was started.

## Evolution

**Fig. 1:**
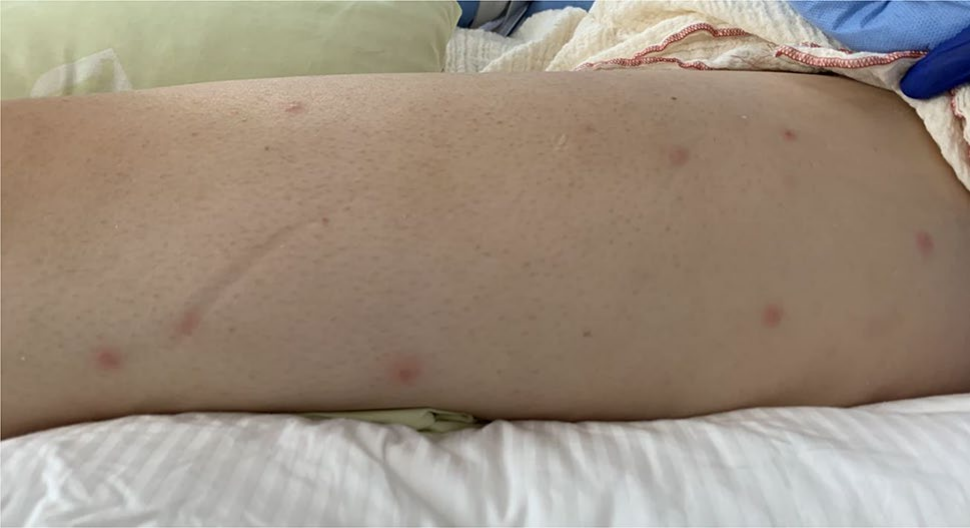
Several red papules measuring 5 mm in diameter on the left leg, which appeared at the time of neutrophil recovery

In initial blood cultures (1/4 bottles), *Capnocytophaga sputigena* (1/4 bottle) and *Streptococcus pneumoniae* (1/4 bottle) grew. At 5 days after admission, while the patient was still neutropaenic, bilateral tonsillectomy and abscess evacuation were performed because of progression of tonsillitis with new tonsillar abscesses, despite adequate antibiotic therapy. On the following day (6 days after admission), however, the patient remained septic. Because of suspected mediastinitis based on fat stranding and multiple prominent mediastinal lymph nodes documented on a CT scan, a sternotomy was carried out with mediastinal debridement, thymectomy and mediastinal lymphadenectomy as well as an explorative left cervicotomy with medio-jugular lymphadenectomy. Mediastinitis was confirmed intraoperatively by the presence of precardial fluid collection with gas inclusions. Blood drawn on this day was cultured, as were intraoperative biopsies of both tonsils and medio-jugular lymph nodes, revealing *Candida albicans.* Treatment with anidulafungin (200 mg as loading dose, followed by 100 mg/d) was started. Follow-up blood cultures were sterile. Transthoracic echocardiography showed no sign of endocarditis. However, her low-grade fever persisted. Bone marrow biopsy showed hypocellular bone marrow, suggestive of toxic effects. Under stimulation with granulocyte-colony stimulating factor (G-CSF) 30 million IU/d for 6 days, neutrophils increased above the threshold level of 500/µL on Day 11 after admission. On the same day, the patient developed several red papules measuring 5 mm in diameter on both arms, legs (Fig. 1), her thorax and her abdomen. Cultures of skin biopsies were sterile. Histopathology showed superficial necrotic epidermis; periodic acid–Schiff (PAS) staining did not indicate any fungal elements.

Fourteen days after admission, the patient was again highly febrile (up to 41 °C) and septic, and CRP increased again (above 340 mg/L). A CT scan revealed new subcutaneous abscess collection overlying the manubrium sterni and multiple hypodense subcapsular lesions in the spleen, measuring 10–18 mm in diameter, but without hepatic lesions. Liver function tests were normal. A second sternotomy with extensive debridement was performed, and *C. albicans* grew in cultures of the biopsies from the mediastinum and sternum. In addition, chronic disseminated candidiasis (CDC) was suspected, and methyl prednisolone at a dose of 1 mg/kg was added to the regimen. Within 2 days, the patient’s condition improved dramatically, and her fever peaks abated in parallel with a decrease in CRP. After 2 weeks of anidulafungin, antifungal therapy was switched to fluconazole (800 mg/d loading dose followed by 200 mg/d), which was intended for at least 1 year to treat *Candida* sternal osteomyelitis. After 2 weeks, corticosteroids were slowly tapered. The patient was discharged after 7 weeks, without antibiotic therapy, and followed up as an outpatient. Important labour parameters in the course of the disease are summarized in Table 1. Abdominal CT scan was not repeated, but an ultrasound at 10 weeks after her initial presentation revealed the presence of multiple new (more than ten) round hypoechogenic lesions in the liver, measuring 5–10 mm in diameter, together with prominent lymph nodes in the hepatic hilum, supporting the diagnosis of CDC. The known lesions in the spleen were still visible but smaller. Corticosteroids were tapered over 6 months; there was no recurrence of fever.

**Table 1:** Labour parameters in the course of the disease

	Admission	+ 5 days	+ 6 days	+ 11 days	+ 14 days	+ 16 days	Discharge
Leucocytes (G/L)	0.25	0.35	0.56	5.2	33.1	36.1	17.4
Neutrophils (G/L)	0	0.02	0.02	2.4	26.2	26.7	
Haemoglobin (g/L)	107	75	78	76	79	83	106
Thrombocytes (G/L)	458	572	649	451	222	313	578
CRP (mg/L)	336	294	333	293	343	142	110
AST (U/L)	17	19		52	34		20
ALT (U/L)	14			35		59	25
ALP (U/L)	57			235			125
GGT (U/L)	77	79			843		168

+ 5 days: first surgical intervention; + 6 days: candidaemia; + 11 days: end of the neutropaenia; + 14 days: begin with corticosteroids

*AST* aspartate aminotransferase, *ALT* alanine aminotransferase, *ALP* alkaline phosphatase, *GGT* gamma-glutamyl transferase

## Discussion

To our knowledge, this is the first reported case of CDC in a patient with metamizole-induced neutropaenia. During neutropaenia, the patient mainly had bacterial and fungal tonsillitis and mediastinitis with sternal osteomyelitis. The candidaemia most likely originated from the tonsillitis, as cultures from the tonsils and medio-jugular lymph nodes grew *C. albicans* in great quantities. In this patient with documented candidaemia, the diagnosis of CDC was based on signs of severe inflammation together with her reduced general condition following immune reconstitution and simultaneous appearance of new hypodense splenic lesions in imaging studies and of skin lesions, suggestive of previously occult haematogenous dissemination.

CDC is a severe complication of disseminated yeast infection that mainly affects the liver and spleen (also known as chronic hepatosplenic candidiasis) and was first described in 1969 [1]. Immunocompromised patients, particularly after profound and prolonged neutropaenia in the context of chemotherapy to treat haematological malignancies, are most at risk [2]. The incidence of CDC has decreased in recent decades with the systematic use of antifungal prophylaxis with azoles in patients with acute leukaemia. CDC develops in two stages: first, during neutropaenia, digestive translocation may occur across the disrupted mucosal barrier of the gastrointestinal tract followed by haematogenous dissemination of *Candida* spp., which may be asymptomatic; second, shortly after neutrophil recovery, a “paradoxical” reaction is observed, with recurrent high fever, poor general condition, right upper quadrant discomfort and nausea. This second phase is thought to be due to overreaction of the immune system to disseminated yeast infection, similar to immune reconstitution inflammation syndrome (IRIS) [2–4]. Despite antifungal therapy, fever and inflammation may persist for weeks [2, 3, 5]. Alkaline phosphatase and other liver enzymes may also be elevated. MRI has much higher sensitivity than ultrasonography or CT for detecting micro-abscesses, which are most frequently localized in the liver and spleen [4]. In most cases, histopathology reveals epithelioid granulomas (which are the hallmark of CDC) but also necrosis and micro-abscesses. Yeasts are detected in biopsies in fewer than 50% of cases. In more than 80% of cases, blood cultures are negative at the time of CDC diagnosis [2, 4].

IRIS is well described in HIV patients with severe immune deficiency. With the start of antiretroviral therapy and strengthening of the immune system, opportunistic infections may be unmasked or paradoxically worsen with systemic or local inflammatory reactions at the site of infection. Many different pathogens have been associated with the development of IRIS. The leading pathogens in the case of HIV infection include *Mycobacterium tuberculosis, Mycobacterium avium complex*, *Cryptococcus neoformans*, *Pneumocystis jirovecii*, Cytomegalovirus, Herpes simplex virus and Human herpes virus 8 [6]. Paradoxical inflammatory syndromes have also been described in HIV-uninfected patients following treatment for tuberculosis, particularly lymph node tuberculosis, known as paradoxical upgrading reactions (PURs) [7]. These reactions can also occur during the course of various invasive fungal diseases (IFDs) after reversal of immune deficiency [4]. In all cases, the cornerstone is an increased inflammatory response following immune recovery. IRIS is now believed to arise from an unregulated Th1/Th17 response leading to increased production of interferon-ɣ [4]. Interferon-ɣ favours classical activation of macrophages to transform into M1-phenotype macrophages, which secrete large amounts of pro-inflammatory cytokines such as IL-1β, TNFα, IL-12, IL-18 and IL-23 [4, 8]. Excess interferon-ɣ produced by Th1 cells, neutrophils or activated macrophages will also promote differentiation of monocytes towards macrophages, activate their phagocytic activity and stimulate granuloma formation [4].

Due to adverse effects (mostly immunosuppressive effects), the use of corticosteroids in IFD-IRIS is still controversial. However, addition of corticosteroids to antifungal therapy in CDC-associated IRIS was shown to be safe and to lead to rapid clinical improvement, especially cessation of fever, in all reported cases [5, 9–15]. It also allowed for the planned treatment for the underlying haematological disease to continue. This is particularly important because delaying chemotherapy or bone marrow transplantation has been associated with poor prognosis. IDSA guidelines for the management of candidiasis recommend considering short-term treatment with nonsteroidal anti-inflammatory drugs or corticosteroids for patients who have debilitating persistent fever. The suggested daily dosage of corticosteroids is 0.5–1 mg/kg of oral prednisone [16]. In most reported cases, the duration of steroid treatment was several weeks or months, with slow tapering of the dose [3, 5].

In our patient, neutropaenia was most likely induced by metamizole. This drug is an old antipyretic and analgesic drug with a mechanism of action that is still not entirely understood [17]. Neutropaenia is a rare adverse event unrelated to the dose or duration of treatment. In general, neutropaenia can cause serious infectious complications with fatal outcomes in up to 16% of cases [18]. Based on World Health Organization (WHO) and Swiss pharmacovigilance data, 52% of the international and 33% of the Swiss metamizole-associated reported haematological adverse drug reactions occurred within 7 days, and the average daily dose was within the range recommended by the manufacturer [17]. In Switzerland, the incidence rate of metamizole-induced neutropaenia between 2006 and 2012 was 0.46–1.63 cases per million person-days of use [17].

In summary, we report a case of chronic disseminated candidiasis following neutropaenia induced by metamizole. This highlights a rare but potentially severe adverse effect of metamizole. We discuss the similarity between CDC and IRIS and emphasize the role of adjuvant corticosteroids. We believe that in this case, in accordance with the literature, the use of corticosteroids resulted in significant improvement of the patient's symptoms, allowing for a shorter hospital stay and a faster return to daily life. We, therefore, encourage systematic use of corticosteroids for this condition.

## Data Availability

Further patient data cannot be shared due to legal and ethical reasons.
